# μ-Oxalato-κ^4^ *O* ^1^,*O* ^2^:*O* ^1′^,*O* ^2′^-bis­[aqua­(2,2′-bipyridine-κ*N*)(nitrato-κ^2^ *O*,*O*′)lead(II)]

**DOI:** 10.1107/S1600536812040196

**Published:** 2012-09-29

**Authors:** Gang-Hong Pan, Jin-Niu Tang, Zhong-Jing Huang, Long Li, Chun-Mei Zhang

**Affiliations:** a College of Chemistry and Chemical Engineering, Guangxi University for Nationalities, Nanning 530006, People’s Republic of China

## Abstract

The title compound, [Pb_2_(C_2_O_4_)(NO_3_)_2_(C_10_H_8_N_2_)_2_(H_2_O)_2_], was synthesized hydro­thermally. The binuclear complex mol­ecule is centrosymmetric, the inversion centre being located at the mid-point of the oxalate C—C bond. The Pb^II^ ion is hepta­coordinated by the O atom of one water mol­ecule, two oxalate O atoms, two nitrate O atoms and two 2,2′-bipyridine N atoms, forming an irregular coordination environemnt. Inter­molecular O—H⋯O hydrogen bonds between water mol­ecules and oxalate and nitrate ions result in the formation of layers parallel to (010). π–π inter­actions between pyridine rings in adjacent layers, with centroid–centroid distances of 3.584 (2) Å, stabilize the structural set-up.

## Related literature

For general background to this class of compounds, see: Fan & Zhu (2006 ▶); Hamilton *et al.* (2004 ▶); Hagrman & Zubieta (2000 ▶); Li *et al.* (2002 ▶).
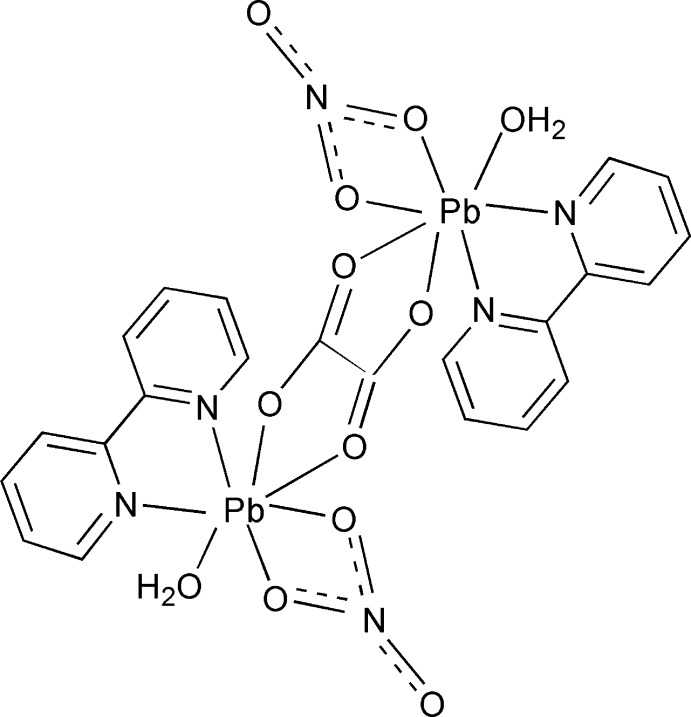


## Experimental

### 

#### Crystal data

[Pb_2_(C_2_O_4_)(NO_3_)_2_(C_10_H_8_N_2_)_2_(H_2_O)_2_]
*M* *_r_* = 974.82
Monoclinic, 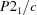

*a* = 9.5791 (19) Å
*b* = 20.6330 (14) Å
*c* = 6.7649 (15) Å
β = 91.687 (1)°
*V* = 1336.5 (4) Å^3^
*Z* = 2
Mo *K*α radiation
μ = 12.66 mm^−1^
*T* = 296 K
0.29 × 0.28 × 0.26 mm

#### Data collection

Bruker SMART CCD diffractometer
Absorption correction: multi-scan (*SADABS*; Bruker, 2003 ▶) *T*_min_ = 0.120, *T*_max_ = 0.137
7096 measured reflections
2336 independent reflections
2082 reflections with *I* > 2σ(*I*)
*R*_int_ = 0.038

#### Refinement

*R*[*F* ^2^ > 2σ(*F* ^2^)] = 0.031
*wR*(*F* ^2^) = 0.082
*S* = 1.07
2336 reflections
190 parameters
H-atom parameters constrained
Δρ_max_ = 2.68 e Å^−3^
Δρ_min_ = −1.19 e Å^−3^

### 

Data collection: *SMART* (Bruker, 2003 ▶); cell refinement: *SAINT* (Bruker, 2003 ▶); data reduction: *SAINT*; program(s) used to solve structure: *SHELXS97* (Sheldrick, 2008 ▶); program(s) used to refine structure: *SHELXL97* (Sheldrick, 2008 ▶); molecular graphics: *DIAMOND* (Brandenburg, 2009 ▶); software used to prepare material for publication: *SHELXTL* (Sheldrick, 2008 ▶).

## Supplementary Material

Crystal structure: contains datablock(s) I, global. DOI: 10.1107/S1600536812040196/wm2667sup1.cif

Structure factors: contains datablock(s) I. DOI: 10.1107/S1600536812040196/wm2667Isup2.hkl

Additional supplementary materials:  crystallographic information; 3D view; checkCIF report

## Figures and Tables

**Table 1:** Hydrogen-bond geometry (Å, °)

*D*—H⋯*A*	*D*—H	H⋯*A*	*D*⋯*A*	*D*—H⋯*A*
O7—H7*A*⋯O5^i^	0.85	2.23	2.875 (7)	133
O7—H7*B*⋯O3^ii^	0.85	2.16	2.912 (8)	148

Symmetry codes: (i) 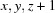; (ii) 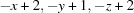.

**Fig. 1.**
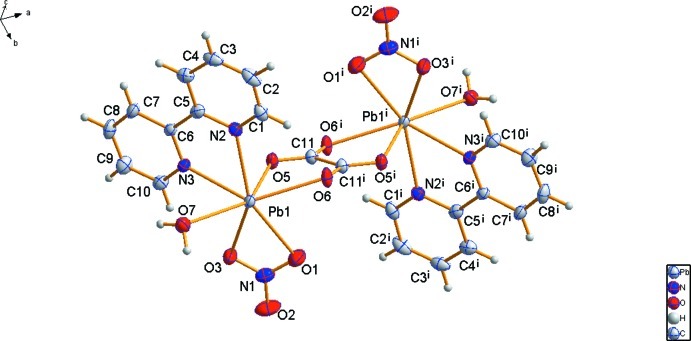
The coordination environment around PbII in the title compound with the atom-labeling scheme. Displacement ellipsoids for non-hydrogen atoms are drawn at the 30% probability level. [Symmetry code: (i) -x+1, -y+1, -z+1.]

**Fig. 2.**
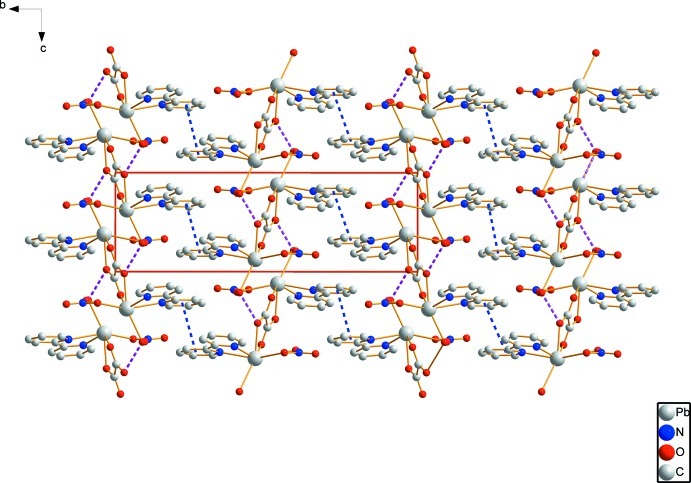
O—H···O hydrogen bonding (purple dashed lines) and π—π stacking interactions (blue dashed lines) in the structure of the title compound. All H atoms were omitted for clarity.

## supplementary crystallographic information

### Comment

Complexes containing Pb^II^ (Fan & Zhu *et al.*, 2006) are interesting because of the variety of their structures and their potential applications, especially in environmental protection, e.g heavy metal removal. It is known that the introduction of chelating ligands such as 2,2'-bipyridine causes the passivation of metal sites via the N donors of the organic groups and may induce new structural evolution (Hamilton *et al.*, 2004; Hagrman & Zubieta, 2000; Li *et al.*, 2002).

In the title binuclear lead(II) compound, [Pb_2_(C_2_O_4_)(NO_3_)_2_(H_2_O)_2_(C_10_H_8_N_2_)_2_], a centrosymmetric molcule is present, with the centre of symmetry at the mid-point of the C—C oxalate bond (Fig. 1). The Pb^II^ ion is hepta-coordinated in an irregular fashion by one water molecule, two nitrate oxygen atoms, two oxalate oxygen atoms, and two nitrogen atoms from 2,2'-bipyridine. The supramolecular assembly in the title compound is completed by O—H···O hydrogen bonds between the coordinating water molecules and oxalate and nitrate O atoms (Table 1), resulting in the formation of layers parallel to (010) (Fig. 2). The structure is further extended by π—π stacking interactions between 2,2'-bipyridine molecules of adjacent layers. They overlap with a centroid-to-centroid distance of 3.584 (2) Å.

### Experimental

A mixture of oxalic acid (0.0634 g, 0.5 mmol), 2,2'-bipyridine (0.0781 g, 0.5mmol), Pb(NO_3_)_2_ (0.3312 g, 1mmol), NaOH (0.0400 g, 1mmol), water (10 ml) and ethanol (5 ml) was placed in a Parr Teflon-lined stainless steel vessel (25 cm^3^). The vessel was then sealed and heated at 403 K for 3 days. Afterwards the mixture was slowly cooled to room temperature, colorless block-shaped crystals of the complex were obtained. Elemental analysis calculated (%_wt_): C 27.11; H 2.07; N 8.62; found (%_wt_): C 27.06; H 2.03; N 8.51.

### Refinement

H atoms were positioned geometrically and refined as riding atoms, with C—H = 0.93 Å and *U*_iso_(H) = 1.2*U*_eq_(C) for aromatic H atoms. H atoms of the water molecule were located in a difference Fourier map and refined as riding, with O—H = 0.85 Å and with *U*_iso_(H) = 1.2*U*_eq_(O). The maximum remaining electron density is found 0.97 Å from Pb1, and the minimum density 0.91 Å from the same atom.

### Crystal data

**Table:**

[Pb_2_(C_2_O_4_)(NO_3_)_2_(C_10_H_8_N_2_)_2_(H_2_O)_2_]	*F*(000) = 908
*M**_r_* = 974.82	*D*_x_ = 2.422 Mg m^−^^3^
Monoclinic, *P*2_1_/*c*	Mo *K*α radiation, λ = 0.71073 Å
Hall symbol: -P 2ybc	Cell parameters from 3490 reflections
*a* = 9.5791 (19) Å	θ = 2.3–28.3°
*b* = 20.6330 (14) Å	µ = 12.66 mm^−^^1^
*c* = 6.7649 (15) Å	*T* = 296 K
β = 91.687 (1)°	Block, colorless
*V* = 1336.5 (4) Å^3^	0.29 × 0.28 × 0.26 mm
*Z* = 2	

### Data collection

**Table:**

Bruker SMART CCD diffractometer	2336 independent reflections
Radiation source: fine-focus sealed tube	2082 reflections with *I* > 2σ(*I*)
Graphite monochromator	*R*_int_ = 0.038
phi and ω scans	θ_max_ = 25.0°, θ_min_ = 2.0°
Absorption correction: multi-scan (*SADABS*; Bruker, 2003)	*h* = −11→10
*T*_min_ = 0.120, *T*_max_ = 0.137	*k* = −24→24
7096 measured reflections	*l* = −7→8

### Refinement

**Table:**

Refinement on *F*^2^	Primary atom site location: structure-invariant direct methods
Least-squares matrix: full	Secondary atom site location: difference Fourier map
*R*[*F*^2^ > 2σ(*F*^2^)] = 0.031	Hydrogen site location: inferred from neighbouring sites
*wR*(*F*^2^) = 0.082	H-atom parameters constrained
*S* = 1.07	*w* = 1/[σ^2^(*F*_o_^2^) + (0.035*P*)^2^ + 5.1328*P*] where *P* = (*F*_o_^2^ + 2*F*_c_^2^)/3
2336 reflections	(Δ/σ)_max_ = 0.001
190 parameters	Δρ_max_ = 2.68 e Å^−^^3^
0 restraints	Δρ_min_ = −1.19 e Å^−^^3^

### Special details

**Table:**

Geometry. All esds (except the esd in the dihedral angle between two l.s. planes) are estimated using the full covariance matrix. The cell esds are taken into account individually in the estimation of esds in distances, angles and torsion angles; correlations between esds in cell parameters are only used when they are defined by crystal symmetry. An approximate (isotropic) treatment of cell esds is used for estimating esds involving l.s. planes.
Refinement. Refinement of F^2^ against ALL reflections. The weighted R-factor wR and goodness of fit S are based on F^2^, conventional R-factors R are based on F, with F set to zero for negative F^2^. The threshold expression of F^2^ > 2sigma(F^2^) is used only for calculating R-factors(gt) etc. and is not relevant to the choice of reflections for refinement. R-factors based on F^2^ are statistically about twice as large as those based on F, and R- factors based on ALL data will be even larger.

### Fractional atomic coordinates and isotropic or equivalent isotropic displacement parameters (Å^2^)

**Table:**

	*x*	*y*	*z*	*U*_iso_*/*U*_eq_	
Pb1	0.68880 (3)	0.537173 (12)	0.87652 (4)	0.03036 (13)	
N1	0.7954 (9)	0.3925 (3)	0.8164 (10)	0.0504 (18)	
N2	0.6271 (6)	0.6548 (3)	0.8129 (9)	0.0345 (14)	
N3	0.8863 (6)	0.6061 (3)	0.7555 (8)	0.0317 (13)	
O1	0.6656 (8)	0.4032 (3)	0.7926 (12)	0.0678 (18)	
O2	0.8406 (9)	0.3377 (3)	0.8302 (12)	0.084 (2)	
O3	0.8765 (6)	0.4408 (3)	0.8182 (10)	0.0563 (16)	
O5	0.6731 (5)	0.5291 (2)	0.5190 (8)	0.0393 (13)	
O6	0.5602 (5)	0.4830 (3)	0.2626 (7)	0.0411 (12)	
O7	0.8279 (6)	0.5894 (3)	1.2112 (8)	0.0463 (13)	
H7A	0.8289	0.5600	1.2993	0.056*	
H7B	0.9123	0.5956	1.1805	0.056*	
C1	0.4989 (8)	0.6777 (4)	0.8412 (12)	0.0449 (19)	
H1	0.4275	0.6480	0.8607	0.054*	
C2	0.4664 (9)	0.7424 (4)	0.8432 (13)	0.052 (2)	
H2	0.3755	0.7562	0.8633	0.062*	
C3	0.5712 (9)	0.7861 (4)	0.8148 (12)	0.049 (2)	
H3	0.5521	0.8303	0.8155	0.058*	
C4	0.7051 (9)	0.7647 (4)	0.7851 (12)	0.0408 (18)	
H4	0.7772	0.7940	0.7661	0.049*	
C5	0.7308 (8)	0.6978 (3)	0.7841 (10)	0.0332 (16)	
C6	0.8707 (7)	0.6707 (3)	0.7405 (10)	0.0294 (15)	
C7	0.9824 (8)	0.7097 (4)	0.6843 (12)	0.0425 (18)	
H7	0.9715	0.7544	0.6737	0.051*	
C8	1.1074 (8)	0.6813 (4)	0.6453 (13)	0.051 (2)	
H8	1.1828	0.7067	0.6091	0.061*	
C9	1.1214 (8)	0.6160 (4)	0.6595 (13)	0.047 (2)	
H9	1.2056	0.5961	0.6315	0.057*	
C10	1.0100 (8)	0.5801 (4)	0.7156 (11)	0.0384 (17)	
H10	1.0206	0.5354	0.7267	0.046*	
C11	0.5660 (7)	0.5032 (3)	0.4367 (11)	0.0319 (15)	

### Atomic displacement parameters (Å^2^)

**Table:**

	*U*^11^	*U*^22^	*U*^33^	*U*^12^	*U*^13^	*U*^23^
Pb1	0.02946 (19)	0.03118 (19)	0.03045 (19)	−0.00956 (10)	0.00100 (11)	0.00129 (10)
N1	0.066 (5)	0.037 (4)	0.048 (4)	0.001 (4)	0.006 (3)	0.002 (3)
N2	0.032 (3)	0.036 (3)	0.035 (3)	0.003 (3)	0.002 (3)	−0.002 (3)
N3	0.031 (3)	0.029 (3)	0.035 (3)	−0.007 (2)	0.002 (2)	−0.001 (3)
O1	0.063 (5)	0.044 (4)	0.097 (5)	−0.009 (3)	−0.001 (4)	0.006 (4)
O2	0.119 (7)	0.032 (4)	0.101 (6)	0.018 (4)	0.009 (5)	0.004 (4)
O3	0.043 (3)	0.042 (3)	0.083 (5)	−0.008 (3)	−0.005 (3)	0.006 (3)
O5	0.027 (3)	0.051 (3)	0.040 (3)	−0.011 (2)	0.003 (2)	−0.001 (2)
O6	0.029 (3)	0.065 (3)	0.030 (3)	−0.013 (2)	0.004 (2)	−0.012 (3)
O7	0.040 (3)	0.055 (3)	0.044 (3)	−0.005 (3)	0.003 (2)	0.006 (3)
C1	0.032 (4)	0.062 (6)	0.041 (5)	0.001 (4)	0.005 (3)	−0.001 (4)
C2	0.043 (5)	0.066 (6)	0.047 (5)	0.023 (4)	0.005 (4)	−0.008 (4)
C3	0.065 (6)	0.039 (5)	0.041 (5)	0.017 (4)	−0.007 (4)	−0.005 (4)
C4	0.049 (5)	0.038 (4)	0.035 (4)	0.006 (4)	−0.001 (3)	−0.002 (3)
C5	0.041 (4)	0.030 (4)	0.028 (4)	0.000 (3)	0.001 (3)	−0.005 (3)
C6	0.034 (4)	0.028 (4)	0.026 (4)	−0.005 (3)	0.000 (3)	0.000 (3)
C7	0.042 (4)	0.036 (4)	0.050 (5)	−0.016 (3)	0.004 (3)	0.004 (3)
C8	0.034 (4)	0.061 (6)	0.058 (6)	−0.019 (4)	0.011 (4)	0.001 (4)
C9	0.035 (4)	0.057 (5)	0.051 (5)	0.000 (4)	0.015 (3)	−0.003 (4)
C10	0.037 (4)	0.036 (4)	0.042 (5)	0.000 (3)	0.006 (3)	−0.004 (3)
C11	0.026 (4)	0.033 (4)	0.037 (4)	−0.001 (3)	0.003 (3)	−0.001 (3)

### Geometric parameters (Å, º)

**Table:**

Pb1—O5	2.425 (5)	C1—C2	1.371 (12)
Pb1—N3	2.522 (5)	C1—H1	0.9300
Pb1—N2	2.532 (6)	C2—C3	1.368 (12)
Pb1—O6^i^	2.572 (5)	C2—H2	0.9300
Pb1—O3	2.718 (6)	C3—C4	1.377 (11)
Pb1—O7	2.809 (6)	C3—H3	0.9300
N1—O2	1.214 (9)	C4—C5	1.401 (10)
N1—O3	1.263 (9)	C4—H4	0.9300
N1—O1	1.269 (10)	C5—C6	1.490 (10)
N2—C1	1.334 (9)	C6—C7	1.401 (9)
N2—C5	1.351 (9)	C7—C8	1.366 (11)
N3—C10	1.336 (9)	C7—H7	0.9300
N3—C6	1.344 (9)	C8—C9	1.358 (12)
O5—C11	1.270 (9)	C8—H8	0.9300
O6—C11	1.249 (9)	C9—C10	1.362 (11)
O6—Pb1^i^	2.572 (5)	C9—H9	0.9300
O7—H7A	0.8500	C10—H10	0.9300
O7—H7B	0.8499	C11—C11^i^	1.554 (13)
			
O5—Pb1—N3	74.93 (17)	C2—C1—H1	118.1
O5—Pb1—N2	83.62 (18)	C3—C2—C1	118.2 (8)
N3—Pb1—N2	65.04 (19)	C3—C2—H2	120.9
O5—Pb1—O6^i^	66.07 (16)	C1—C2—H2	120.9
N3—Pb1—O6^i^	131.97 (18)	C2—C3—C4	120.0 (8)
N2—Pb1—O6^i^	83.30 (19)	C2—C3—H3	120.0
O5—Pb1—O3	80.03 (19)	C4—C3—H3	120.0
N3—Pb1—O3	81.95 (18)	C3—C4—C5	118.8 (8)
N2—Pb1—O3	146.01 (19)	C3—C4—H4	120.6
O6^i^—Pb1—O3	116.03 (18)	C5—C4—H4	120.6
O5—Pb1—O7	147.62 (16)	N2—C5—C4	121.1 (7)
N3—Pb1—O7	72.69 (17)	N2—C5—C6	116.8 (6)
N2—Pb1—O7	82.64 (18)	C4—C5—C6	122.0 (7)
O6^i^—Pb1—O7	140.35 (16)	N3—C6—C7	120.5 (7)
O3—Pb1—O7	95.61 (18)	N3—C6—C5	117.1 (6)
O2—N1—O3	121.0 (8)	C7—C6—C5	122.4 (6)
O2—N1—O1	121.2 (8)	C8—C7—C6	119.1 (7)
O3—N1—O1	117.7 (7)	C8—C7—H7	120.4
C1—N2—C5	118.2 (7)	C6—C7—H7	120.4
C1—N2—Pb1	121.7 (5)	C9—C8—C7	119.8 (7)
C5—N2—Pb1	119.0 (5)	C9—C8—H8	120.1
C10—N3—C6	118.7 (6)	C7—C8—H8	120.1
C10—N3—Pb1	121.2 (5)	C8—C9—C10	118.9 (8)
C6—N3—Pb1	120.0 (4)	C8—C9—H9	120.5
N1—O3—Pb1	99.7 (5)	C10—C9—H9	120.5
C11—O5—Pb1	119.4 (4)	N3—C10—C9	123.0 (7)
C11—O6—Pb1^i^	114.5 (4)	N3—C10—H10	118.5
Pb1—O7—H7A	106.6	C9—C10—H10	118.5
Pb1—O7—H7B	107.0	O6—C11—O5	124.5 (6)
H7A—O7—H7B	106.7	O6—C11—C11^i^	118.5 (8)
N2—C1—C2	123.8 (8)	O5—C11—C11^i^	117.0 (8)
N2—C1—H1	118.1		

Symmetry code: (i) −*x*+1, −*y*+1, −*z*+1.

### Hydrogen-bond geometry (Å, º)

**Table:**

*D*—H···*A*	*D*—H	H···*A*	*D*···*A*	*D*—H···*A*
O7—H7*A*···O5^ii^	0.85	2.23	2.875 (7)	133
O7—H7*B*···O3^iii^	0.85	2.16	2.912 (8)	148

Symmetry codes: (ii) *x*, *y*, *z*+1; (iii) −*x*+2, −*y*+1, −*z*+2.
